# High-Speed Three-Dimensional Scanning Force Microscopy Visualization of Subnanoscale Hydration Structures on Dissolving Calcite Step Edges

**DOI:** 10.1021/acs.nanolett.4c02368

**Published:** 2024-08-26

**Authors:** Kazuki Miyata, Kosuke Adachi, Naoyuki Miyashita, Keisuke Miyazawa, Adam S. Foster, Takeshi Fukuma

**Affiliations:** † Nano Life Science Institute (WPI-NanoLSI), Kanazawa University, Kakuma-machi, Kanazawa 920-1192, Japan; ‡ Division of Electrical Engineering and Computer Science, Kanazawa University, Kakuma-machi, Kanazawa 920-1192, Japan; ¶ Department of Applied Physics, Aalto University, Helsinki FI-00076, Finland

**Keywords:** Three-Dimensional Scanning Force Microscopy, Molecular Dynamics Simulation, Calcite, Crystal Dissolution, Transition Region

## Abstract

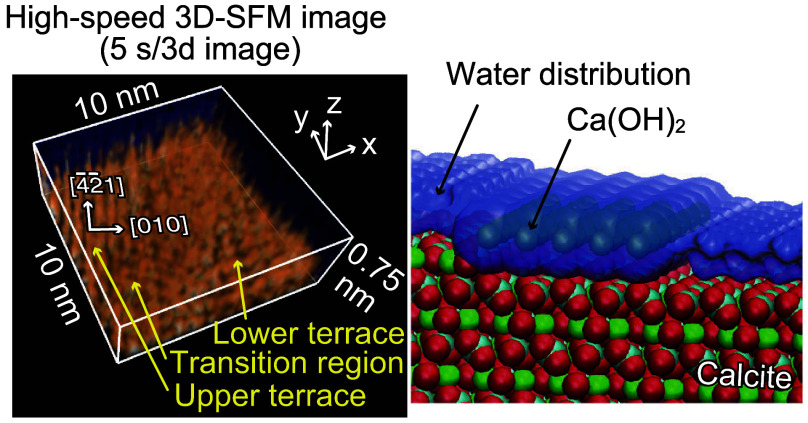

Hydration at solid–liquid interfaces plays an essential role in a wide range of phenomena in biology and in materials and Earth sciences. However, the atomic-scale dynamics of hydration have remained elusive because of difficulties associated with their direct visualization. In this work, a high-speed three-dimensional (3D) scanning force microscopy technique that produces 3D images of solid–liquid interfaces with subnanoscale resolution at a rate of 1.6 s per 3D image was developed. Using this technique, direct 3D images of moving step edges were acquired during calcite dissolution in water, and hydration structures on transition regions were visualized. A Ca(OH)_2_ monolayer was found to form along the step edge as an intermediate state during dissolution. This imaging process also showed that hydration layers extended from the upper terraces to the transition regions to stabilize adsorbed Ca(OH)_2_. This technique provides information that cannot be obtained via conventional 1D/2D measurement methods.

Solid–liquid interfaces are ubiquitous in nature and technology. The interface hydration state plays central roles in a wide range of phenomena, including adhesion, corrosion, wetting,^1,2^ protein folding, stability and recognition,^3,4^ and mineral growth and dissolution.^5,6^ Solid–liquid interface structures and properties have been studied via X-ray and neutron beam analysis techniques;^7−9^ atomic force microscopy (AFM);^10^ and theoretical simulations using molecular dynamics (MD),^11^ kinetic Monte Carlo methods,^12^ and density functional theory.^13^

Nevertheless, the effects of hydration on physical or chemical processes at interfaces remain poorly understood. These phenomena may involve molecular-level dynamic events, including water exchange, dissociative adsorption on solid surfaces, and hydrogen bonding network formation.^14^ The lack of in situ imaging techniques for changes in solid–liquid interfacial structures is challenging. Although AFM is promising, several issues must be resolved. First, AFM offers limited temporal or spatial resolution. High-speed amplitude modulation AFM can visualize dynamic processes at solid–liquid interfaces with imaging rates from 10 to 100 ms/frame.^15^ However, its spatial resolution is typically on the nanoscale. In contrast, frequency modulation AFM (FM-AFM) can visualize atomic-scale structures at solid–liquid interfaces.^16^ Unfortunately, its typical imaging rate is approximately 1 min/frame, which is insufficient for dynamic processes visualization. Additionally, AFM was originally developed to image 2D surface structures rather than 3D structures; therefore, it is difficult to visualize 3D hydration structures directly by using AFM.

To address these shortcomings, we recently increased the FM-AFM speed by approximately 2 orders of magnitude, achieving atomic-resolution imaging in liquids at approximately 0.5 s/frame. This high-speed FM-AFM (HS-FM-AFM) technique allows imaging of interfacial phenomena on a time scale of seconds. One example application is imaging of calcite (CaCO_3_) crystal dissolution in water (Figure 1a,b). Previous research showed that calcite dissolution involves rhombic etch pit formation followed by dissolution at pit edges (Figure 1c, Supplemental Movie 1),^17,18^ but atomistic behavior at the step edges is not yet understood because direct observation is difficult. HS-FM-AFM enabled direct visualization of atomic-scale structural changes at dissolving step edges on calcite crystals (Figure 1d, Supplemental Movie 2).^19^ The images showed the formation of a layer-like structure, termed a transition region (TR), along the step edges with an intermediate height between the upper and lower terrace levels (Figure 1e). Additionally, simulations indicated that the TR was likely a Ca(OH)_2_ monolayer that formed as an intermediate state during calcite dissolution (Figure 1f). The lattice constant along the *c*-axis of bulk Ca(OH)_2_ (i.e., portlandite) is ∼0.48 nm,^20^ which is larger than the calcite step height (∼0.3 nm). However, our simulations indicate that the Ca(OH)_2_ monolayer at the TR has a different, thinner structure.^19^ Furthermore, the Ca^2+^ and OH^–^ ion positions remain relatively stable, indicating that lattice regularity is largely maintained, while the OH^–^ ion orientations fluctuate. Therefore, the TR crystal structure differs from that of bulk Ca(OH)_2_, and its surface is below the upper terrace. This is typically but not always consistent with the appearance of FM-AFM images, as shown in Figure 1d.^21^ FM-AFM is so sensitive that the tip is typically scanned on one of the hydration layers formed above a hydrophilic surface; the apparent height difference between the TR and the terraces in an FM-AFM image thus varies with differences in the imaging conditions, including the feedback set point and tip properties. We previously reported variations where the TR appeared to be at an intermediate height between the upper and lower terraces, at the upper terrace height, or below the lower terrace height. We also reported their statistical properties,^21^ behaviors in a small etch pit,^22^ and possible roles in step dissolution kinetics.^22^ Furthermore, we found that a TR exists at the step edge not only during the crystal dissolution process but also during the crystal growth process.^23^

**Figure 1:**
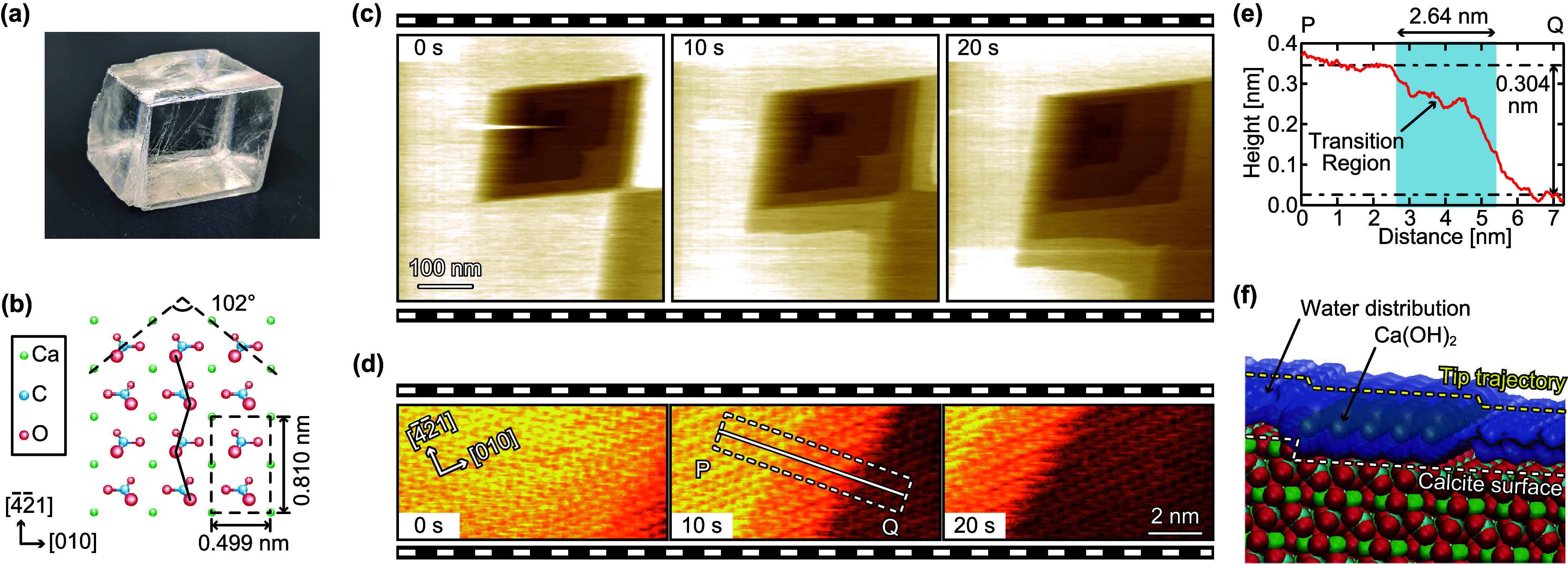
High-speed (HS)-FM-AFM imaging of calcite dissolution. (a) Photograph of the calcite crystal used in the HS-FM-AFM and 3D scanning force microscopy (3D-SFM) experiments. (b) Atom-scale model of the calcite (101̅4) surface. (c) Successive HS-FM-AFM images of etch pits on the calcite (101̅4) surface in water. Imaging rate: 5 s/frame. Pixel size: 500 × 500 pix^2^. Image size: 500 × 500 nm^2^. (d) Successive HS-FM-AFM images of a dissolving step edge on a calcite (101̅4) surface in water. Imaging rate: 1 s/frame. Pixel size: 500 × 250 pix^2^. Image size: 10 × 5 nm^2^. (e) Averaged height profile measured along line P–Q indicated in panel d. The dotted lines around line P–Q indicate the width of the averaging process. (f) Model of a step edge with a transition region (TR) made of Ca(OH)_2_. The white dotted line indicates the calcite surface with a step edge, and the yellow dotted line illustrates the tip trajectory during HS-FM-AFM imaging.

However, the calcite dissolution mechanism postulating Ca(OH)_2_ monolayer formation has not been widely accepted in the mineralogy or crystal growth research field because comparisons between 2D FM-AFM experiments and 3D simulation data are indirect. Assuming the Ca(OH)_2_ layer’s existence, we calculated the 3D hydration structure and converted it to a 3D force map, a 3D frequency shift map, and then to a 2D height image and finally compared it with the experimental 2D FM-AFM images.^19^ The excellent agreement between these images indicated our model’s validity. However, because several assumptions and expert AFM knowledge are required to convert simulated 3D hydration structures into 2D height images, the reliability and clarity of these arguments are insufficient for general acceptance. Additionally, the mechanism enabling stable TR formation at the step edges remains unclear. Ca(OH)_2_ is unstable in bulk neutral solutions or on flat terraces,^24^ suggesting that TR stabilization involves interactions with step edges. Typical TR widths are approximately several nanometers, indicating that direct interactions between step edges and adsorbed ions cannot be the sole stabilization mechanism.^21^ Another possibility involves indirect interactions via a hydration structure. From our simulations and FM-AFM results,^19,21^ a hydration structure formed on an upper terrace extends almost seamlessly over the TR. The favorable energetics associated with generation of this extra hydrogen bonding network may enable wide adsorption layer formation. However, there is no direct evidence for production of this unique hydration structure at step edges from 3D measurements, and this hypothesis is not yet widely accepted.

Direct hydration structure visualization became possible after development of 3D-AFM, which images 3D force distributions at solid–liquid interfaces.^25,26^ In this technique, the AFM tip is scanned vertically as well as laterally, and variations in the force applied to the tip during scanning are recorded to produce 3D force images. During scanning, the tip interacts with surrounding water and surface structures; the resulting force map shows the distribution of the interacting molecules. 3D-AFM imaging is primarily used as an extension of one-dimensional (1D) force curve measurements because its implementation is simple (Figure S1a).^27^ However, this 3D imaging requires several minutes to perform because of the complex tip motions, and images can be distorted by tip drift. To permit faster measurements, we developed 3D scanning force microscopy (3D-SFM) (Figure S1b).^28^ In 3D-SFM, force distributions are measured while modulating the vertical tip position using a sinusoidal signal (*z*_m_ in Figure 2b) at a rate that exceeds the tip–sample distance control bandwidth. This produces a smooth scanning trajectory and enables faster scanning without spurious vibrations. This technique has been combined with FM detection to visualize subnanoscale 3D hydration structures on minerals,^28,29^ biomolecules,^30,31^ biomaterials,^32,33^ and polymers.^34^ However, conventional 3D-SFM imaging rates are limited to approximately 1 min/3D image, which is too slow for most dynamic events, including atomic-scale phenomena at dissolving calcite step edges.

**Figure 2:**
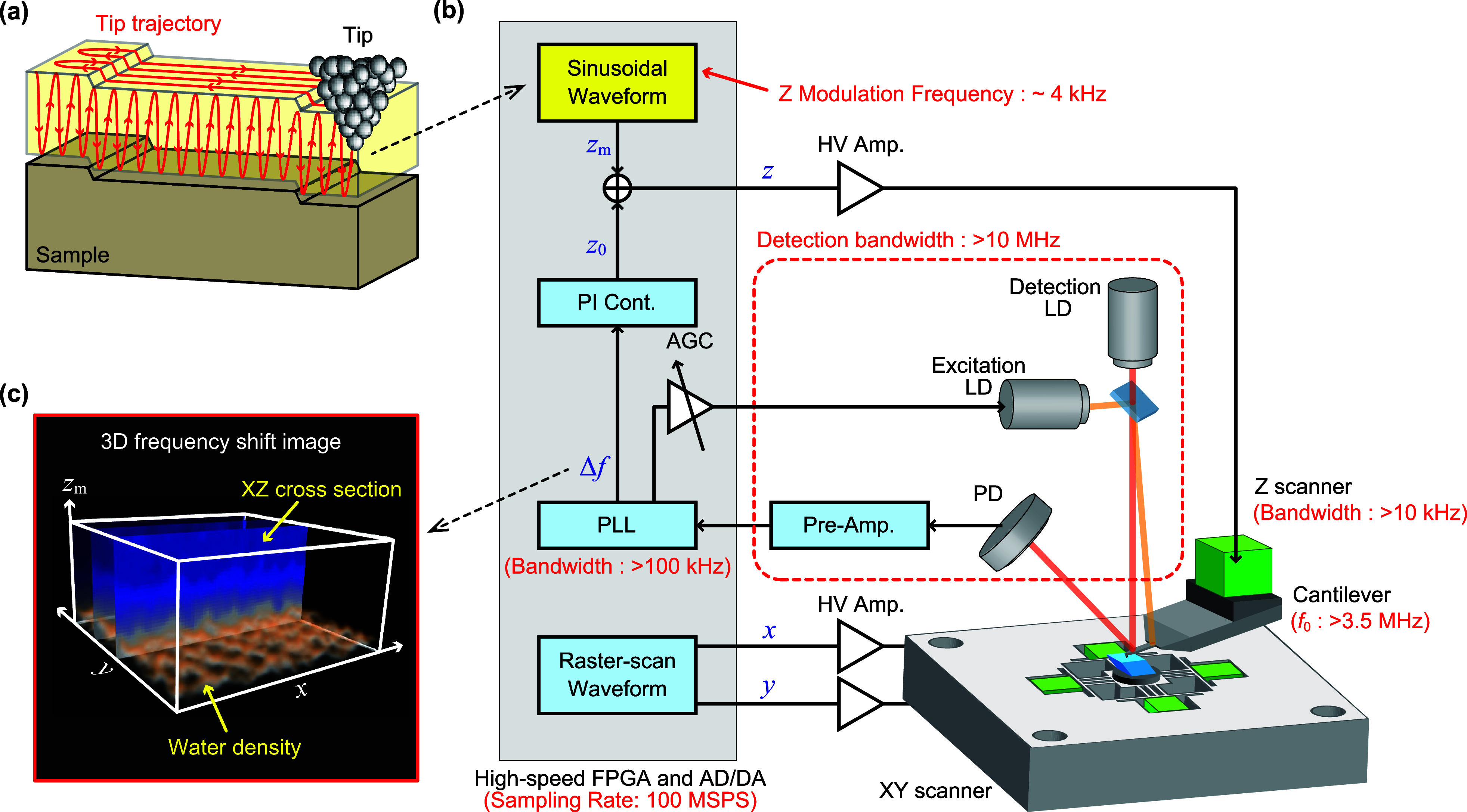
Setup for the newly developed HS-3D-SFM system and a 3D-SFM image obtained by using this system. (a) Diagram showing the basic principle of 3D-SFM. (b) Experimental setup for the newly developed HS-3D-SFM system. (c) HS-3D-SFM image of a calcite (101̅4) surface in water. Imaging rate: 5 s/3D image. Pixel size: 100 × 100 × 256 pix^3^. Image size: 5 × 5 × 1.5 nm^3^.

This work combines HS-FM-AFM with 3D-SFM to develop high-speed 3D-SFM (HS-3D-SFM) with a rate of 1.6 s/3D image, which is over ten times faster than conventional 3D-SFM. Using this technique, direct 3D hydration structure imaging around moving step edges of calcite crystals during dissolution in water was demonstrated with subnanoscale resolution. Using 3D-SFM images and MD simulations, molecular-scale 3D hydration structures at step edges were elucidated, and firm evidence supporting the aforementioned hypothesis concerning TR formation was obtained. These data serve to improve our understanding of the calcite dissolution mechanism at the atomic level.

To increase 3D-SFM’s speed without compromising its atomic-scale resolution, a number of conditions must be met. First, the measurement bandwidth (*B*) must be increased while maintaining force resolution (*F*_min_) of less than 10–100 pN. Second, rapid *z*_m_ signal synchronization with the high-speed XY scan is required. Finally, fast cantilever frequency shift signal (Δ*f*) recording is necessary. To satisfy the first condition, significant effort was applied to improving several key AFM elements, including ultrasmall cantilevers with a megahertz-order resonance frequency (*f*_0_) in liquids,^35^ a low-noise wide-band frequency shift detector,^36−38^ a cantilever deflection sensor,^39−41^ a cantilever photothermal excitation system,^35,41^ and Z-tip and XY-sample scanners.^42,43^ If the cantilever’s vibrational amplitude is small enough for the force gradient to be considered constant, *F*_min_ is given by
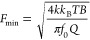
1where *k*_B_, *T*, and *B* are Boltzmann’s constant, absolute temperature, and the measurement bandwidth, respectively. In 3D-SFM, *B* should be sufficiently large compared with the frequency (*f*_mod_) of the *z*_m_ signal. Assuming that frequency shift signals up to the 10th harmonic of *f*_mod_ should be acquired to obtain clear 3D images, *B* must equal 10*f*_mod_. Additionally, *f*_mod_ is determined by the time required to generate each 3D image (*T*_img_) and by the numbers of X and Y pixels (*P*_*x*_ and *P*_*y*_) that must be recorded. Assuming that the tip is raster-scanned laterally, *T*_img_ will equal 2*P*_*x*_*P*_*y*_/*f*_mod_. Using these equations, *B* and *T*_img_ for commercial high-*f*_0_ cantilevers and the required *F*_min_ values were estimated (Table S1). These values indicate that the smallest cantilever used here (USC-F5-k30, Nanoworld) will allow 3D-SFM imaging at 1.5 s/3D image while maintaining *F*_min_ of 25 pN.

To achieve this acquisition rate, a 3D-SFM function was implemented in the HS-FM-AFM.^19^ Changes for this implementation included addition of a *z*_m_ signal generator, an adder to calculate *z*_m_ + *z*_0_ and a fast recorder for Δ*f* (Figure 2b). To meet the second and third conditions, these components were implemented in a field-programmable gate array (FPGA) board (PXIe-7966R, National Instruments) with 100 MSPS analog input/output interfaces (NI-5781, National Instruments) (Figure 2b). Next, we discuss the main issues that were overcome during the implementation.

To satisfy the second condition (*z* and *xy* scan synchronization at high scan rates), we considered several issues. First, *z* modulation must not begin after the tip–sample distance regulation is established to avoid tip and sample damage. Instead, we should turn on the *z* modulation at far from the surface and then make a gentle approach to establish feedback control. Second, *xy* scans should not begin immediately after a user request, but when the tip reaches the highest *z* position in a *z* modulation cycle for synchronization. Third, small mismatches between the ends of the line scan and the *z* modulation cycle should be compensated at the end of each scan line by poling. Finally, the most important and difficult point is to minimize the latency from the *z* modulation signal output to the recording of Δ*f* signal to cancel its influence on the force curves by postprocessing. This requirement was satisfied by the hardware improvements, analog input/output interfaces with low in-to-out latency (∼400 ns), and low-latency digital signal processing algorithms (e.g, phase-locked loop circuit, proportinal-integral controllers for amplitude and tip–sample distance) implemented in a high-speed FPGA. We achieved overall latency of ∼7.6 μs with the USC-F5-k30, enabling 3D-SFM imaging with an acceptable synchronization errors at 1.6 s/3D image.

To satisfy the third condition (fast recording), we enhanced the data transfer rates between FPGA components and the data storage device (Figure S2). For flexibility and expandability, we aimed to record nine channels simultaneously (*z* position, deflection, amplitude, frequency, phase, dissipation, three AUX channels) for each approach and retract *z* profile and for each forward and backward *x* scan with a pixel size of 256 × 256 × 256 pix^3^. This requires a data transfer and recording rate of 2.4 GB/s. Hard disk drives (HDDs) (50–120 MB/s) and solid-state drives (200–500 MB/s) have insufficient recording rates. Therefore, we used 24 parallel HDDs with RAID0 configurations (HDD-8266, National Instruments) for a 3.6 GB/s maximum recording rate. Data transfer from the FPGA is performed with a 3.2 GB/s direct access memory (DMA) transfer rate to the host PC through a PXIe chassis (NI-1071, National Instruments, 3 GB/s) and an MXI-Express Gen-3 ×16 interface (PXIe-8398, National Instruments, 4 GB/s). Practical issues were considered during implementation. For DMA transfer, we selected an appropriate block size based on the imaging speed to avoid overhead effects. We transferred data recorded during a *z* modulation cycle or an *xz* scan at one time for slow (>10 s/3D image) or fast (<10 s/3D image) imaging speeds, respectively. After data transfer to the host PC, the multichannel data were unbundled and selected for saving or viewing. These processes should be implemented independently in parallel for high throughput.

Using this system, 3D-SFM imaging of calcite–water interfacial structures was performed at 5 s/3D image (Figure 2c). From the image size (100 × 100 × 256 pix^3^) and USC-F5-k30 cantilever characteristics, the system’s *F*_min_ was approximately 33 pN. This value matched typical 3D-SFM imaging requirement (10–100 pN). Despite the high imaging rates obtained in these trials, subnanoscale force distributions corresponding to the hydration structures were visualized.

Dynamic hydration structure changes during calcite dissolution were visualized using USC-F5-k30 and AC55 (Olympus) cantilevers with approximate *f*_0_ values of 3.5 and 1.5 MHz, respectively, in an aqueous environment (Table S1). Although the USC-F5-k30 provided a lower *F*_min_, its electron-beam deposited tip should be replaced with a stronger tip for atomic-resolution imaging.^44^ The AC55 provided a higher *F*_min_ but permitted atomic-level resolution without tip modification at high scan speeds (Table S1).

Using the AC55, the hydration structure near a step edge on the calcite surface was imaged at 5 s/3D image (Figure 3a, Supplemental Movie 3). At 0 s, the surface showed a uniform subnanoscale contrast pattern (region i). Subsequently, region ii (width: approximately 3 nm) appeared from the right and gradually moved left; region iii then appeared. Structural pattern differences are evident from the top view of the 3D image obtained at 40 s (Figure 3b, Figure S3). Height data acquired simultaneously with the 3D-SFM image showed that the height difference between regions i and iii was approximately 0.3 nm, representing a single step height on the calcite (101̅4) surface (Figure S4). Regions i and iii were upper and lower terraces, respectively, while region ii represented a TR at an intermediate height between them.

**Figure 3:**
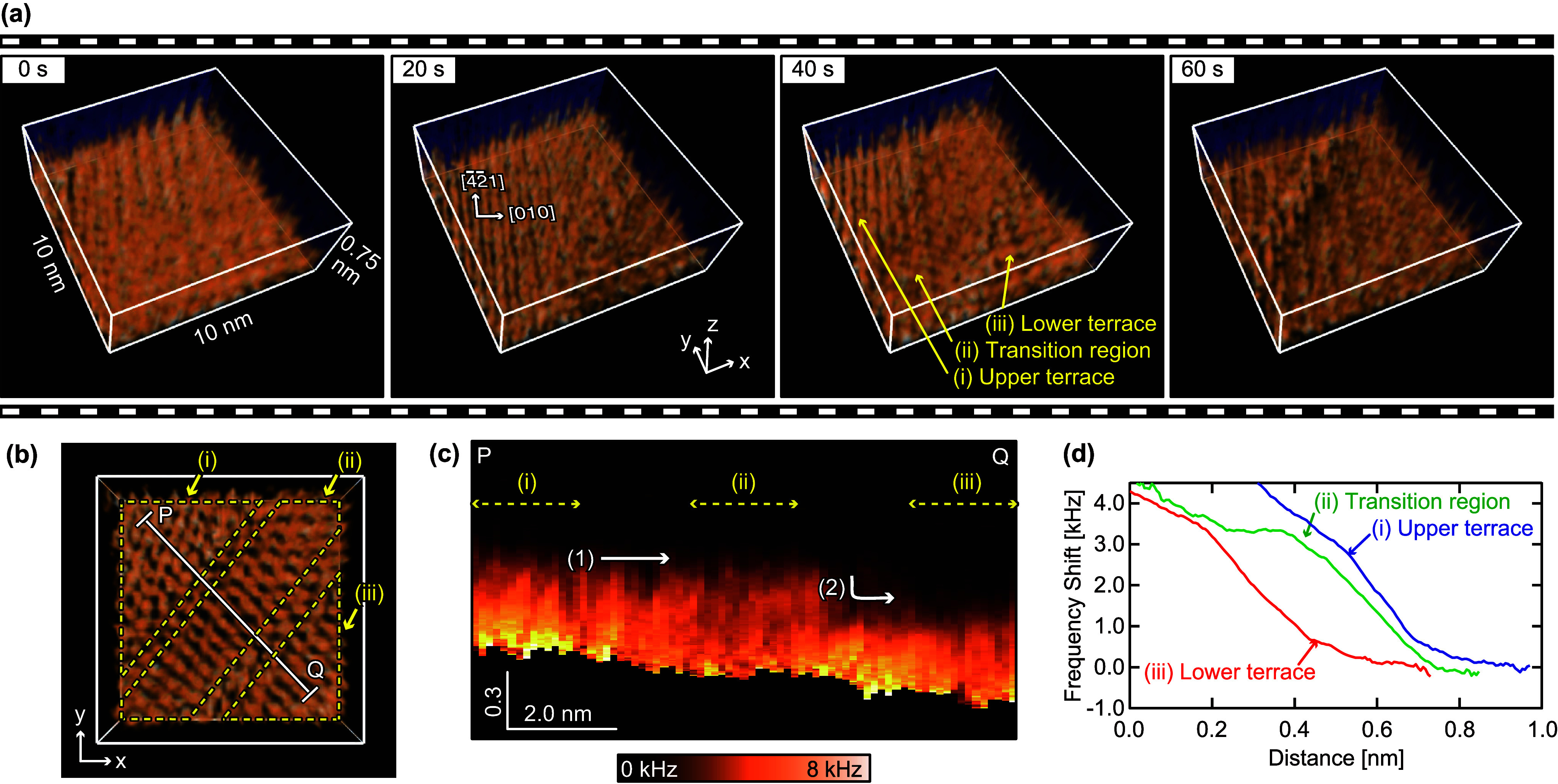
HS-3D-SFM imaging of a dissolving calcite step edge. (a) Successive HS-3D-SFM images acquired near a dissolving step edge on a calcite (101̅4) surface in water. Imaging rate: 5 s/3D image. Pixel size: 100 × 100 × 128 pix^3^. Image size: 10 × 10 × 0.75 nm^3^. (b) Top view of the HS-3D-SFM image obtained at 40 s, as shown in panel a. (c) Averaged vertical cross section measured along line P–Q, as indicated in panel b. The widths of the perpendicular bars at both ends of line P–Q show the averaging width. The Z position was corrected using a 2D height image acquired simultaneously. (d) Δ*f* curves averaged over the areas indicated by the yellow dotted lines labeled i–iii in panel c.

The vertical cross section (Figure 3c) along line P–Q in Figure 3b was analyzed. This cross section was placed across regions i–iii to compare Δ*f* distributions corresponding to each region’s hydration structures. Regions i and iii had similar structures, while the region ii structure differed, reflecting terrace and TR hydration structure variations. Comparison of regions i and ii showed that the region ii hydration structure was thicker, and the upper hydration structure edges were almost the same height (arrow 1). However, the upper edge position was significantly lower in region iii (arrow 2). This is reasonable, because the lower and upper terraces are expected to have identical hydration structures. The Δ*f Z* profiles (Figure 3d) averaged over the width indicated by yellow arrows in Figure 3c showed that regions i and iii had almost equivalent profiles, except for a height difference of a single step thickness. The *Z* profile for region ii differed significantly, suggesting the TR’s hydration structure was different. This *Z* profile variation is likely to affect the 2D height images of the step edges acquired by FM-AFM in the constant Δ*f* mode. We previously reported variations in FM-AFM images of TRs, which appeared as protrusions or depressions depending on the Δ*f* set point for the tip–sample distance regulation.^21^

It is difficult to distinguish experimentally between Δ*f* values induced by a crystal surface and that induced by water above it. Therefore, best practice involves detailed comparisons between experiments and simulations to determine the most plausible models. We previously made such comparisons at calcite/water interfaces, and atomistic models of the surface and hydration structures and their imaging mechanisms are well-established.^45,46^ For TR, we previously compared 2D FM-AFM results with 3D models of the surface and hydration structures from simulations. The uncertainty when 2D experiments are linked to 3D theoretical models highlighted the need for this HS-3D-SFM study. The 3D hydration structure obtained by HS-3D-SFM is compared with theoretical predictions later.

We improved *F*_min_ using the USC-F5-k30 cantilever, and the hydration structure near a dissolving calcite step edge was visualized at 1.6 s/3D image (Figure 4a, Figure S5); this is more than ten times faster than previous imaging speeds for 3D subnanoscale hydration structure measurements.^47−49^ The 3D-SFM images were compared with a simulated water density map. The vertical cross section (Figure 4b) along line P–Q in Figure 4a indicated that the upper edge positions of the hydration structures on the upper terrace and TR were almost equal, whereas that on the lower terrace was significantly lower (Figure 4b). These features matched those in Figure 3, confirming their reproducibility.

**Figure 4:**
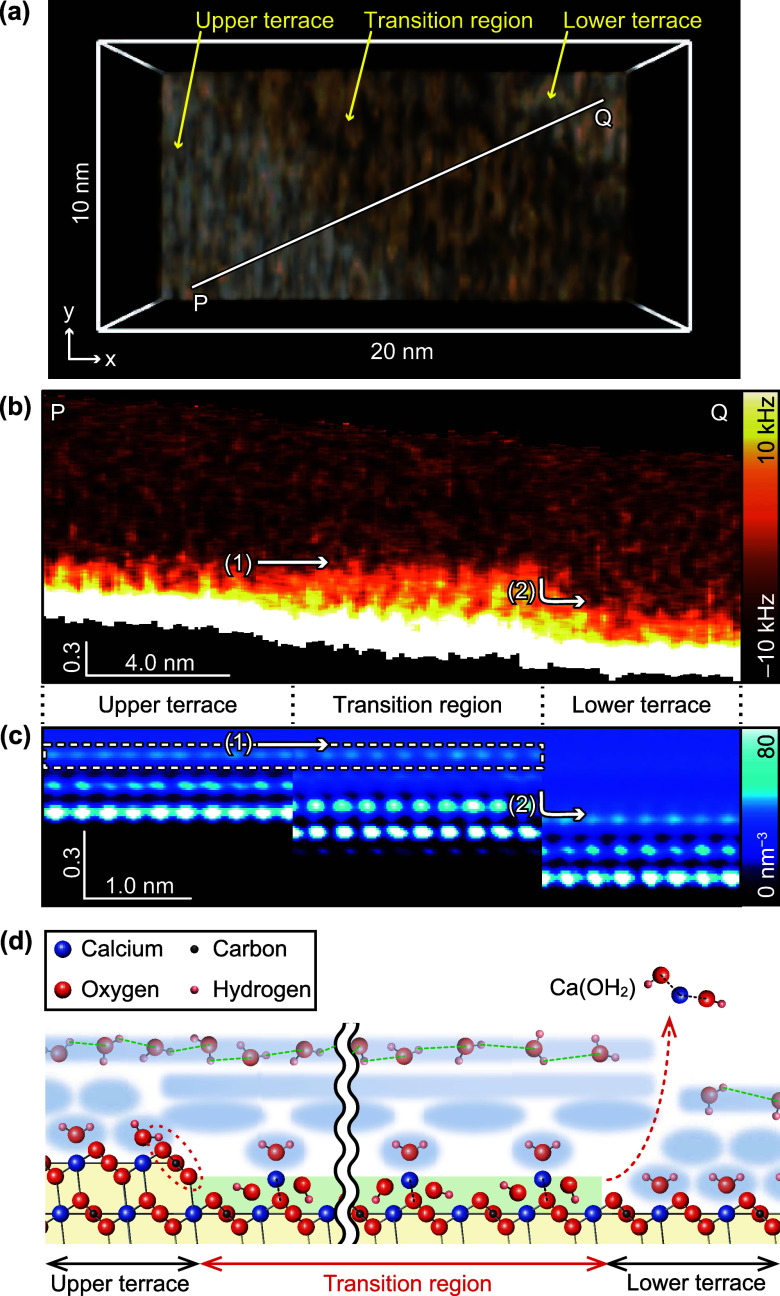
Comparison between HS-3D-SFM image and MD simulation results. (a) HS-3D-SFM image of a dissolving step edge on a calcite (101̅4) surface in water. Imaging rate: 1.6 s/3D image. Pixel size: 128 × 32 × 256 pix^3^. Image size: 20 × 10 × 2 nm^3^. (b) Vertical cross section acquired along line P–Q as indicated in panel a. The Z position has been corrected based on a 2D height image that was acquired simultaneously. (c) MD simulation of a calcite (101̅4) surface in water with a TR composed of Ca(OH)_2_. The [4̅41] projection of the water density map was obtained by averaging the simulated distribution over each unit cell area. (d) Atomic-scale model of a step edge with a TR made of Ca(OH)_2_ and a hydrogen bonding network that extends from the upper terrace to the TR.

The experimental results were consistent with the water density map from MD simulations (Figure 4c), which showed formation of multiple hydration layers on the terraces and TR. The topmost hydration layers on the upper terrace and TR occurred at the same height, whereas the lower terrace layer was approximately 0.3 nm lower. This agreement between experimental data and simulations validates the simulation model in which the TR was modeled as a Ca(OH)_2_ monolayer formed at the step edge (Figure 4d).

In our previous HS-FM-AFM and MD simulation study, we proposed that a hydration layer extended from the upper terrace to the TR.^19^ However, this was difficult to confirm using 2D observations. The HS-3D-SFM images clearly show the 3D distribution predicted via simulations, supporting the existence of an extended hydration layer. The effect of this layer on TR formation can thus be discussed.

Previous HS-FM-AFM results showed that each TR typically has a width of several nm.^21^ The adsorption structure’s stability was also confirmed via MD simulations,^19^ although Ca(OH)_2_ is unstable in bulk neutral solutions or an atomically flat terraces. The extended hydration layer formation may therefore explain why stable TRs appeared only at step edges. Specifically, Ca(OH)_2_ layer formation at step edges could extend each upper terrace hydration layer to the TR. The associated extended hydrogen bonding network would be energetically favored. Previous HS-FM-AFM images showed molecular-scale stripes extending from an upper terrace to a TR, confirming hydration layer continuity (Figure S6).^21^ The extended layer’s energetic favorability would decrease as the distance from the step edge increased. Additionally, exceptionally thick hydration structure formation on a TR would be energetically unfavorable. Therefore, Ca(OH)_2_ layer growth would cease at a width where these factors are balanced.

For further insights into TR structures, the TR should be imaged using an alternative technique. High-resolution lateral force microscopy (LFM) is potentially applicable to these measurement. Hydration force measurements at a mica/water interface via torsional resonance AFM were demonstrated.^50^ Combining a force detection scheme with HS-3D-SFM may provide additional TR structure information because LFM is sensitive to different interactions than FM-AFM with a vertical cantilever vibration.

An HS-3D-SFM system was developed and visualized the formation of extended hydration layers above moving step edges during calcite dissolution in water. Exceptionally fast 3D imaging with atomic-scale resolution was achieved at 1.6 s/3D image. Based on this extended hydration layer formation, possible TR formation mechanisms and factors determining the TR width were proposed. In conventional atomic-scale analyses of solid–liquid interfacial phenomena, 3D models from MD simulations are compared with 2D HS-FM-AFM images. However, HS-3D-SFM allows for real-space and real-time observations of 3D interfacial structures and direct 3D comparisons between experimental data and simulations. The proposed methodology provides an effective approach to investigating atomic-scale mechanisms of interfacial phenomena.

## Supporting Information Available

Materials and Methods; supplementary figures and table (PDF)
Supplemental Movie 1: Successive HS-FM-AFM images of etch pits on a calcite (101̅4) surface in water; Imaging rate: 5 s/frame; three of these images were selected for the main text and are presented in Figure 1c (MP4)
Supplemental Movie 2: Successive HS-FM-AFM images of a dissolving step edge on a calcite (101̅4) surface in water; imaging rate: 1 s/frame; three of these images were selected for the main text and are presented in Figure 1d (MP4)
Supplemental Movie 3: Successive HS-3D-SFM images obtained near a dissolving step edge on a calcite (101̅4) surface in water; imaging rate: 5 s/3D image; four of these images were selected for the main text and are presented in Figure 3a (MP4)
Input files for the Large-scale Atomic/Molecular Massively Parallel Simulator (LAMMPS) used to run the MD simulation; includes “run.in” and “calcite-hydroxide.500000.start.gz”; the former describes the simulation settings and the parameters of the Buckingham potential, and the latter contains the structural information (ZIP)
