# Supplementary material for: High-Speed Three-Dimensional Scanning Force Microscopy Visualization of Subnanoscale Hydration Structures on Dissolving Calcite Step Edges
Source: Nano Lett. 2024 Aug 26;24(35):10842–9. doi: 10.1021/acs.nanolett.4c02368 (PMC11378286; doi:10.1021/acs.nanolett.4c02368)
Supplement: Supplementary file 1 — nl4c02368_si_001.pdf [file nl4c02368_si_001.pdf]

Supporting Information for

High-speed three-dimensional scanning force microscopy visualization of subnanoscale hydration structures on dissolving calcite step edges

Kazuki Miyata,[1,2*] Kosuke Adachi,[2] Naoyuki Miyashita,[2] Keisuke Miyazawa,[1,2]
Adam S. Foster[1,3†] and Takeshi Fukuma[1,2‡]

[1] Nano Life Science Institute (WPI-NanoLSI), Kanazawa University, Kakuma-machi, Kanazawa 920-1192, Japan
[2] Division of Electrical Engineering and Computer Science, Kanazawa University, Kakuma-machi, Kanazawa 920-1192, Japan
[3] Department of Applied Physics, Aalto University, Helsinki FI-00076, Finland

Correspondence to:
* k-miyata@staff.kanazawa-u.ac.jp
† adam.foster@aalto.fi
‡ fukuma@staff.kanazawa-u.ac.jp

This PDF file includes:

1. Materials and Methods
2. Supplementary Figures and Table
3. References

1. Materials and Methods

HS-FM-AFM and HS-3D-SFM experiments

The present study employed a calcite substrate (Crystal Base Co., Ltd.), with dimensions of 5 × 5 × 3 mm^3, which was glued to a sample holder. Immediately after cleavage of the substrate, 50 μL of Milli-Q water was dropped onto the sample surface. In the dropped water, we performed HS-FM-AFM and HS-3D-SFM imaging at room temperature with two different commercially available cantilevers. In the case of the experiments for which results are shown in Figs. 1c-d and 3a-c, an AC55 cantilever (Olympus) was used. This cantilever had a typical spring constant, k, quality factor, Q, and resonance frequency, f_0, of 80 N/m, 10 and 1.5 MHz, respectively, in an aqueous environment. The experiments for which data are presented in Figs. 2c and 4a used a USC-F5-k30 cantilever (Nanoworld) having k, Q and f_0 values in an aqueous environment of 30 N/m, 10 and 3.5 MHz, respectively. To eliminate the contaminants on the tip surface, both tips were coated with a 15 nm silicon film using a DC sputter coater (K575XD, Emitech).[51] Further information such as firmware/software code, circuit diagrams and mechanical drawings are available from the corresponding author upon request.

MD simulations

The detailed methods for the MD simulation were previously reported elsewhere.[45,52] Thus, here we only describe them briefly. All simulations were performed using the MD code large-scale atomic/molecular massively parallel simulator,[53] and analysis was performed visually using visual molecular dynamics or numerically by bespoke code using the Python library MD analysis.[54,55] For this system, the same force field is used for the MD simulations as in the previous work.[19] In that work, we tested different setups with several species for investigating the origins of the TR and found calcium hydroxide as the most likely model.

2. Supplementary Figures and Table

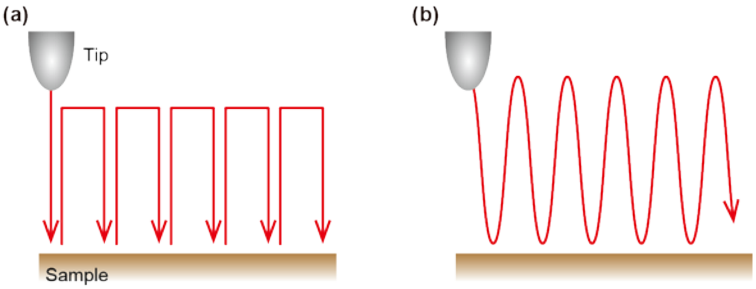

Figure S1. Basic principles of different 3D-AFM modes. (a) Extension of 1D force curve measurements. (b) 3D-SFM, in which the vertical position of the tip is modulated by a sinusoidal signal more faster than the bandwidth of the tip-sample distance control.

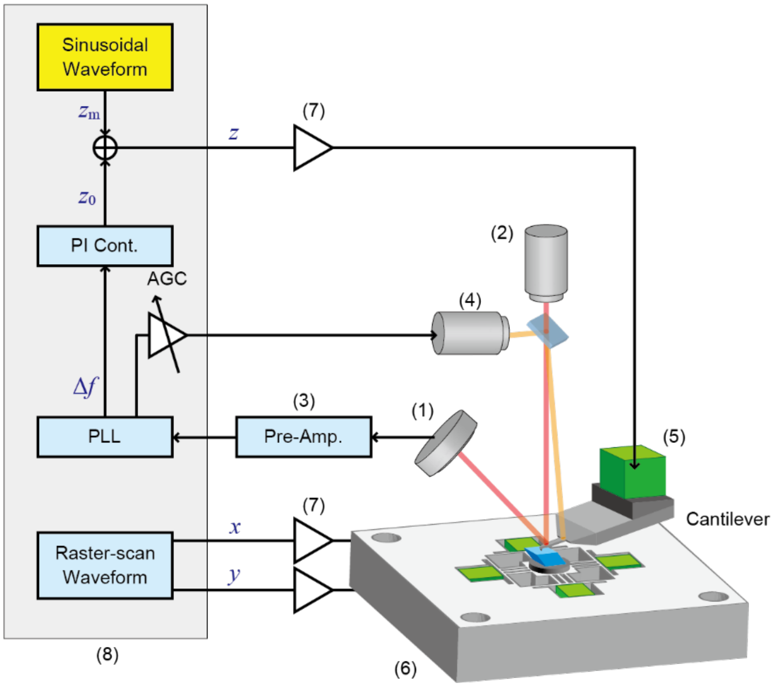

Parts list

No.	Name	Product no., Manufacturer	Ref.
(1)	Photodetector	S6695-01, Hamamatsu Photonics	39-41
(2)	Detection laser	#54-025, Edmund Optics	
	(Laser driver)	IP500, Thorlabs	
(3)	Pre-Amp.	Custom design	
(4)	Excitation laser	57ICS010/SP/HS, Melles Griot	35,41
	(Laser driver)	(Accompanying above)	
(5)	Z scanner	Custom design	42,43
	(Piezo actuator)	PL033, PI Ceramics	
(6)	XY scanner	Custom design	
	(Piezo actuator)	AE0203D04F, NEC Tokin	
(7)	HV-Amp.	Custom design	
(8)	FPGA board	PXIe-7966R, National Instruments	36-38
	(AD/DA interface)	NI-5781, National Instruments	
	(PXIe chassis)	NI-1071, National Instruments	
	(MXI express Gen 3 × 16 interface)	PXIe-8398, National Instruments	
	(parallel 24 HDDs)	HDD-8266, National Instruments	

Figure S2. (Left) The experimental setup for the newly developed HS-3D-SFM system. (Right) Part number and manufacturer of the key components in the AFM head and controller with references to the corresponding papers.

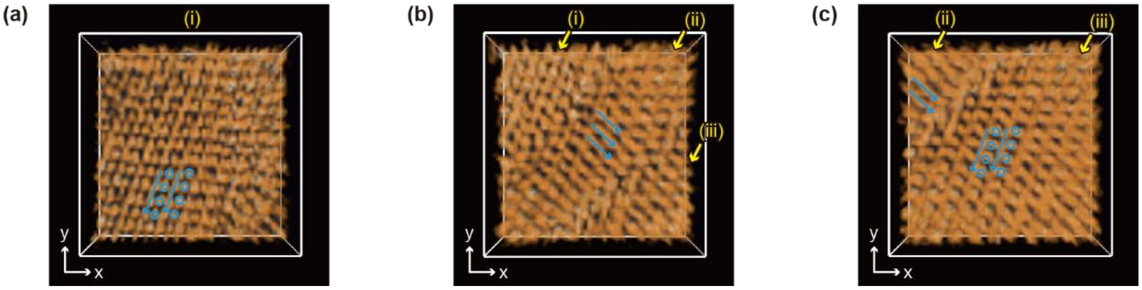

Figure S3. Three snapshots of the HS-3D-SFM images selected from Supplemental Movie 3. (a) Before the step edge appeared in the imaging area. The entire area of the image is on (i) the upper terrace. (b) When the step edge is imaged at the center (the same as Figure 3(b)). (i), (ii) TR and (iii) lower terrace are visualized. (c) After the step edge almost disappeared from the imaging area. Most of the imaging area is on (iii), except for (ii) in the upper left corner. Blue circles and arrows indicate characteristic features commonly observed in (a) and (b). These images show a clear similarity between (i) and (iii), and their difference from (ii).

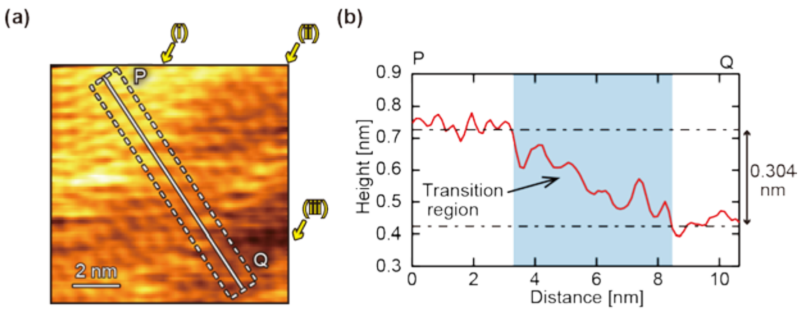

Figure S4. Height image obtained simultaneously with the 3D-SFM image shown in Fig. 3a. (a) Height image obtained at 40 s. (b) Averaged height profile acquired along line P–Q as indicated in (a). The dotted lines around line P–Q indicate the width of the averaging.

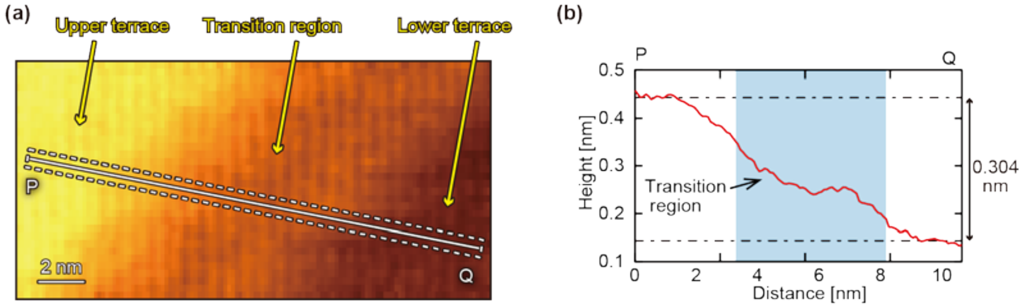

Figure S5. Height image obtained simultaneously with the 3D-SFM image in Fig. 4a. (a) Height image. (b) Averaged height profile acquired along line P–Q as indicated in (a). The dotted lines around line P–Q indicate the width of the averaging.

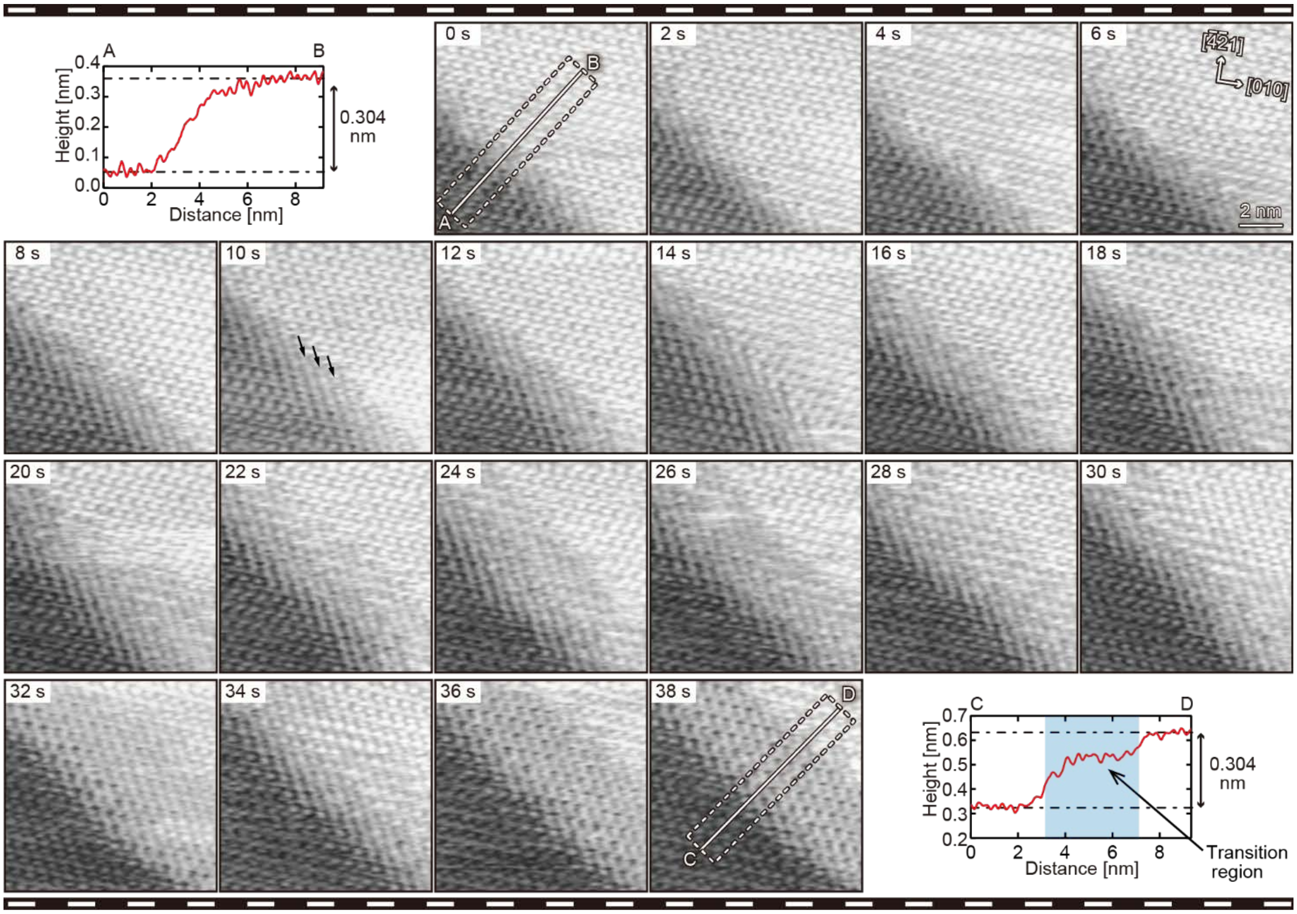

Figure S6. Molecular-scale stripes extending from the upper terrace to the transition region. Successive HS-FM-AFM images of a dissolving step edge on a calcite (10$\bar{1}$4) surface in water and average height profiles acquired along lines A–B and C–D as indicated in the images obtained at 0 and 38 s. Imaging rate: 2 s/frame. Pixel size: 500 × 500 pix^2. Image size: 10 × 10 nm^2. Arrows in the image obtained at 10 s point out molecular-scale stripes.

Table S1. Estimated measurement bandwidths and minimum 3D imaging times for different cantilever types.

Cantilever	f_0 [kHz]	k [N/m]	Q	F_{min} = 10 pN		F_{min} = 25 pN	
				B [kHz]	T_{img} a) [s/3D-image]	B [kHz]	T_{img} a) [s/3D-image]
Conventional type (NCH, Nanoworld)	150	40	10	0.711	281	4.45	45.0
Small type 1 (AC55, Olympus)	1500	80	10	3.56	56.2	22.2	9.00
Small type 2 (USC-F5-k30, Nanoworld)	3500	30	10	22.1	9.04	138	1.45

a) Minimum 3D-imaging time was calculated for an area of 100 × 100 pix^2 assuming that B equals $10f_{mod}$.

3. References

(51) Akrami, S. M. R.; Nakayachi, H.; Watanabe-nakayama, T.; Asakawa, H.; Fukuma, T., Significant improvements in stability and reproducibility of atomic-scale atomic force microscopy in liquid. *Nanotechnology* **2014**, 25, 455701.

(52) Reischl, B.; Watkins, M.; Foster, A. S., Free Energy Approaches for Modeling Atomic Force Microscopy in Liquids. *J. Chem. Theory Comput.* **2013**, 9, 600-608.

(53) Plimpton, S., Fast Parallel Algorithms for Short-Range Molecular Dynamics. *J. Comp. Phys.* **1995**, 117, 1-19.

(54) Humphrey, W.; Dalke, A.; Schulten, K., VMD: visual molecular dynamics. *J. Mol. Graph. Model.* **1996**, 14, 33-38.

(55) Michaud-Agrawal, N.; Denning, E. J.; Woolf, T. B.; Beckstein, O., MDAnalysis: a toolkit for the analysis of molecular dynamics simulations. *J. Comput. Chem.* **2011**, 32, 2319-2327.
